# Causal Relationship Between Emotional Disorders and Thyroid Disorders: A Bidirectional Two‐Sample Mendelian Randomization Study

**DOI:** 10.1002/brb3.70252

**Published:** 2025-01-19

**Authors:** Jiaying Fan, Kai Zhou, Cuiwen Yu

**Affiliations:** ^1^ Department of Endocrinology Ningbo Hospital of Traditional Chinese Medicine Affiliated to Zhejiang Chinese Medical University Ningbo Zhejiang China

**Keywords:** causal relationship, emotional disorders, Mendelian randomization study, thyroid disorders

## Abstract

**Introduction:**

The interplay between emotional disorders and thyroid disorders has been subject to numerous observational studies, which have consistently reported associations but have failed to establish clear causal links due to the multifactorial etiology and influences. We conducted a bidirectional two‐sample Mendelian randomization (MR) analysis to explore the genetic causal association between emotional disorders and thyroid disorders.

**Methods:**

We employed several methods, including inverse‐variance weighted (IVW), weighted median, weighted mode, and MR Egger regression. Additionally, sensitivity analyses were conducted using MR‐Egger, MR Pleiotropy Residual Sum and Outlier (MR‐PRESSO), Cochran's Q, and leave‐one‐out methods.

**Results:**

IVW results showed negative causal relationships between bidirectional emotional disorders and hypothyroidism, toxic single thyroid nodules in thyrotoxicosis, and hyperthyroidism/toxicity. Additionally, there was a positive causal relationship between anxiety disorders and hypothyroidism. IVW results of reverse MR analysis estimates revealed a positive causal relationship between hypothyroidism, autoimmune thyroiditis, and recurrent or chronic depression. Additionally, there was a negative causal relationship between hyperthyroidism/toxicity and bipolar disorder.

**Conclusion:**

This bidirectional two‐sample MR study preliminarily reveals a complex, bidirectional causal relationship between emotional disorders and thyroid disorders, particularly highlighting the role of thyroid dysfunction in the development of certain emotional disorders and vice versa.

## Graphical abstract

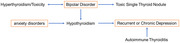

## 1 Introduction

Thyroid disorders constitute a diverse and polymorphic endocrine dysregulation syndrome, primarily including hyperthyroidism, hypothyroidism, and thyroid nodules (Connelly, Park, and LaFranchi 2022). Clinical features vary widely, covering from mild metabolic abnormalities to significant nodular formations and even severe systemic symptoms (Mariani et al., 2021). Globally, these diseases demonstrate an increasing trend, impacting the quality of life for nearly 300 million people, resulting in a significant global burden, thus necessitating a meticulous understanding of the risk factors contributing to their incidence (Bajaj, Salwan, and Salwan 2016). However, the etiology of thyroid disorders is complex, involving multiple dimensions such as genetics, environment, and lifestyle, with genetic factors increasingly gaining attention in recent years.

Emotional disorders such as depression and anxiety are crucial topics in the field of mental health, affecting individuals’ emotional, cognitive, and behavioral manifestations (Dehn and Beblo 2019). These conditions often present with persistent symptoms like low mood, loss of interest, and excessive worry and can lead to social dysfunction when severe. In recent years, mounting research evidence suggests a link between emotional disorders and various physiological mechanisms, including interactions with thyroid function (Gorkhali et al., 2020). Clinical observational studies indicate a close association between thyroid disorders and emotional disorders (Nuñez et al., 2022). Some studies find a higher prevalence of emotional disorders among thyroid disorder patients (Shen et al., 2019), while others report an increased risk of thyroid disorders among individuals with emotional disorders (Zhang et al., 2024). Despite multiple reports of this association, the causal relationship remains unclear as observational studies are susceptible to confounding factors such as psychosocial stress and lifestyle habits, which themselves could be risk factors for either thyroid disorders or emotional disorders.

Mendelian randomization (MR), as a genetics‐based epidemiological method, can more accurately elucidate the causal relationship between thyroid disorders and emotional disorders. MR utilizes naturally occurring genetic variations as “instrumental variables” (IVs) that can theoretically simulate randomized controlled trials, thereby reducing confounding bias and issues of reverse causality prevalent in traditional observational studies (Chen et al., 2024). The two‐sample MR method uses two independent groups of samples: one to detect the relationship between genetic variation and exposure variables and the other to evaluate the relationship between genetic variation and outcome variables (Bowden and Holmes 2019). This separation of samples effectively reduces the interference of confounding factors and reverse causality. Furthermore, the premise of using genetic variation as an instrumental variable is that these variations are unrelated to potential confounding factors and only influence the outcome through the exposure variable, ensuring the accuracy of causal inference. Through rigorous statistical tests and sensitivity analyses, two‐sample MR further examines the validity of assumptions and the robustness of results, ensuring the reliability and scientific nature of the analysis. Sensitivity analysis helps to identify potential biases and assess the results’ sensitivity to various assumptions and parameter changes, making causal relationship assessments more credible (Bowden and Holmes 2019). Therefore, two‐sample MR provides strong evidence for causal relationship evaluation, and its high reliability makes it an important tool in biomedical research.

In this context, this study proposes a two‐sample MR design, acquiring genetic markers for emotional disorders from large‐scale genome‐wide association studies (GWAS) alongside data for thyroid disorders. Through a two‐stage analysis, the study aims to first identify genetic variations associated with emotional disorders and then assess the relationship between these variations and the risk of thyroid disorders. The objective is to overcome the limitations of traditional research and provide evidence to verify whether there is a causal association between emotional disorders and thyroid disorders, offering insights for better understanding their relationship and laying a scientific foundation for future research and clinical practice.

## 2 Materials and Methods

### 2.1 Study Design

**FIGURE 1:**
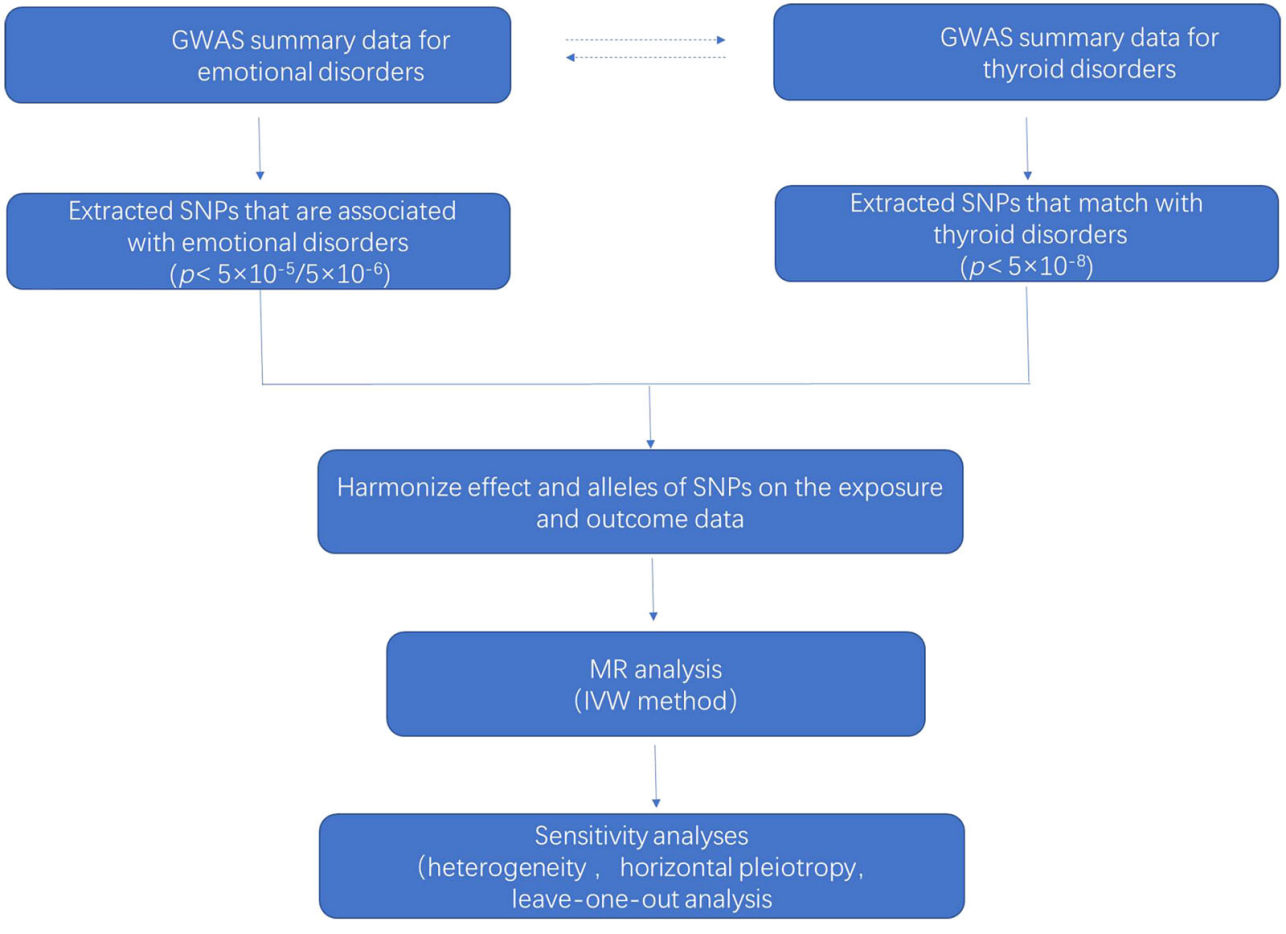
The overall design of this study.

The research design of this study meets the following three key hypotheses (Davies, Holmes, and Davey Smith 2018). Those assumptions are: (1) the relevance assumption (i.e., the genetic variant(s) being used as an instrument for the exposure is associated with the exposure), (2) the independence assumption (i.e., there are no common causes of the genetic variant(s) and the outcome of interest), and (3) the no horizontal pleiotropy assumption (i.e., there is no independent pathway between the genetic variant(s) and the outcome other than through the exposure) (Haycock et al., 2016). Figure 1 depicts the overall design of this study.

### 2.2 Data Source

We utilized a bidirectional two‐sample MR to evaluate the causal association between emotional disorders and thyroid diseases. A schematic overview of the study design is detailed in Figure 1. Specifically, we obtained relevant genetic IVs from the latest and most comprehensive meta‐analysis studies.

The genetic IVs for hypothyroidism (30,155 cases and 379,986 controls), thyroid cancer (1,054 cases and 490,920 controls), and major depressive disorder (7,264 cases and 49,373 controls) were sourced from EBI. The genetic IVs for autoimmune thyroiditis (244 cases and 187,684 controls), nontoxic single thyroid nodule (1,121 cases and 187,684 controls), thyrotoxicosis with toxic single thyroid nodule (110 cases and 214,650 controls), recurrent or chronic depression (19,388 cases and 246,043 controls), and anxiety disorder (27,664 cases and 368,054 controls) were obtained from FinnGen (https://www.finngen.fi/en). Summary statistics for non‐cancer illness code and self‐reported hyperthyroidism/thyrotoxicosis (3,545 cases and 459,388 controls) were collected from the UKB biobank. The genetic IV for bipolar disorder bip2021 (41,917 cases and 371,549 controls) was sourced from MRC‐IEU. All data are publicly available GWAS summary statistics. Hence, no additional ethical approval or informed consent was required. Detailed information regarding the selected GWAS datasets is provided in Table S1.

### 2.3 Selection of Instrumental Variables

We used a multi‐step process to select genetic IVs. We set a *p* value threshold of 5 × 10^−8^ to identify single nucleotide polymorphisms (SNPs) significantly associated with thyroid diseases and emotional disorders (Table S2). The number of SNPs meeting this stringent criterion for bipolar disorder, recurrent or chronic depression, major depression, and anxiety disorders is very limited. Therefore, we relaxed the inclusion criteria to *p* < 5 × 10^−6^ for major depression and anxiety disorders (Xu et al., 2023) and *p* < 5×10^−5^ for bipolar disorder and recurrent or chronic depression (He et al., 2021; Sanderson, Spiller, and Bowden 2021), respectively. Next, we pruned for linkage disequilibrium (LD) between SNPs based on an *R* ^2^< 0.001 and window size = 10,000 kb (Li et al., 2023). In cases where the selected IV was not present in the summary data for the outcome, we looked for a proxy SNP with high LD (*R* ^2^> 0.8) to replace it (Wang et al., 2023). Finally, ensuring that the effect of SNPs on exposure corresponds to the same allele as their effects on outcome is an important step in MR analysis. After matching the outcome, we excluded those with incompatible alleles (e.g., A/C paired with A/G) or being palindromic with intermediate allele frequency (Ji et al., 2024). We utilized these carefully chosen SNPs as the final genetic IVs for the subsequent MR analysis.

Furthermore, we calculated the F‐statistic for each SNP in the IV to assess IV strength, excluding weak instrument bias between the IV and exposure using the following equation: *F* = *R* ^2^ (*N* − 2)/(1 − *R* ^2^), where *R* ^2^ is the proportion of exposure variance explained by the SNP and *N* is the sample size (He et al., 2021). An F‐statistic > 10 indicates the suitability of the IVs, meeting the first assumption of MR analysis (Sanderson, Spiller, and Bowden 2021).

### 2.4 MR Analyses

In this study, we applied multiple complementary approaches, including the inverse variance weighted (IVW), the MR‐Egger regression, the weighted median, and the weighted mode methods, to estimate the causal effects of exposures on outcomes. The IVW method calculates the weighted average effect size by assigning the inverse variance as weights for each SNP. Additionally, the MR‐Egger (Burgess and Thompson 2017), weighted median (Bowden et al., 2016), and weighted mode (Hartwig, Davey Smith, and Bowden 2017) methods were used to test the robustness of the results. The MR‐Egger method considers the presence of an intercept term and can provide accurate estimates of causal effects in the presence of pleiotropy bias. The weighted median method assumes that half of the IVs are valid and analyzes the causal relationship between exposure and outcome. All analyses in this study were conducted using the “Two Sample MR” package in R version 4.0.5. Visualizations were done using scatter plots and sensitivity analysis plots.

### 2.5 Sensitivity Analysis

We assessed heterogeneity among instruments using Cochran's Q test, considering heterogeneity to be low when *P* > 0.05, indicating that the estimates among instruments were randomly distributed and had little impact on the IVW results (Bao et al., 2024). Additionally, we used the MR‐Egger regression method to explore and eliminate the impact of pleiotropy on the estimation of the association due to genetic variation. When the intercept term of the MR‐Egger regression approached zero or was not statistically significant, it suggested the absence of pleiotropy. The “leave‐one‐out” analysis was performed by omitting each instrumental SNP in turn to identify potential heterogeneous SNPs. Furthermore, we employed MR Pleiotropy Residual Sum and Outlier (MR‐PRESSO) to detect and remove potential outliers (SNPs with *p* < 0.05), followed by a re‐estimation of the causal associations to correct for potential pleiotropy (Verbanck et al., 2018).

### 2.6 Bidirectional MR Analysis

We conducted a two‐sample bidirectional MR analysis to explore the reverse causal relationship between thyroid disorders (exposure) and emotional disorders–related diseases (outcome). The steps of bidirectional MR analysis are the same as those of MR analysis.

## 3 Results

### 3.1 Associations of Emotional Disorders–Related Diseases and Thyroid Disorders

In this study, when the corresponding F‐statistic exceeded 10, indicating sufficient correlation strength between IVs and the respective emotional disorder taxon, it was deemed that there was no significant weak instrumental bias (Wei et al., 2023). Fourteen IVs related to severe depression were selected in this study, with a mean F‐statistic of 22.428; 51 bidirectional IVs related to emotional disorders had a mean F‐statistic of 38.097; 13 IVs associated with recurrent or chronic depression had a mean F‐statistic of 36.042; and 61 IVs linked to anxiety disorders had a mean F‐statistic of 24.046. However, in the corresponding MR analysis, 52 SNPs did not match the information in the pooled data, and these unmatched SNPs could not be replaced in the results. Detailed information about the selected IVs is provided in Table S2.

**FIGURE 2:**
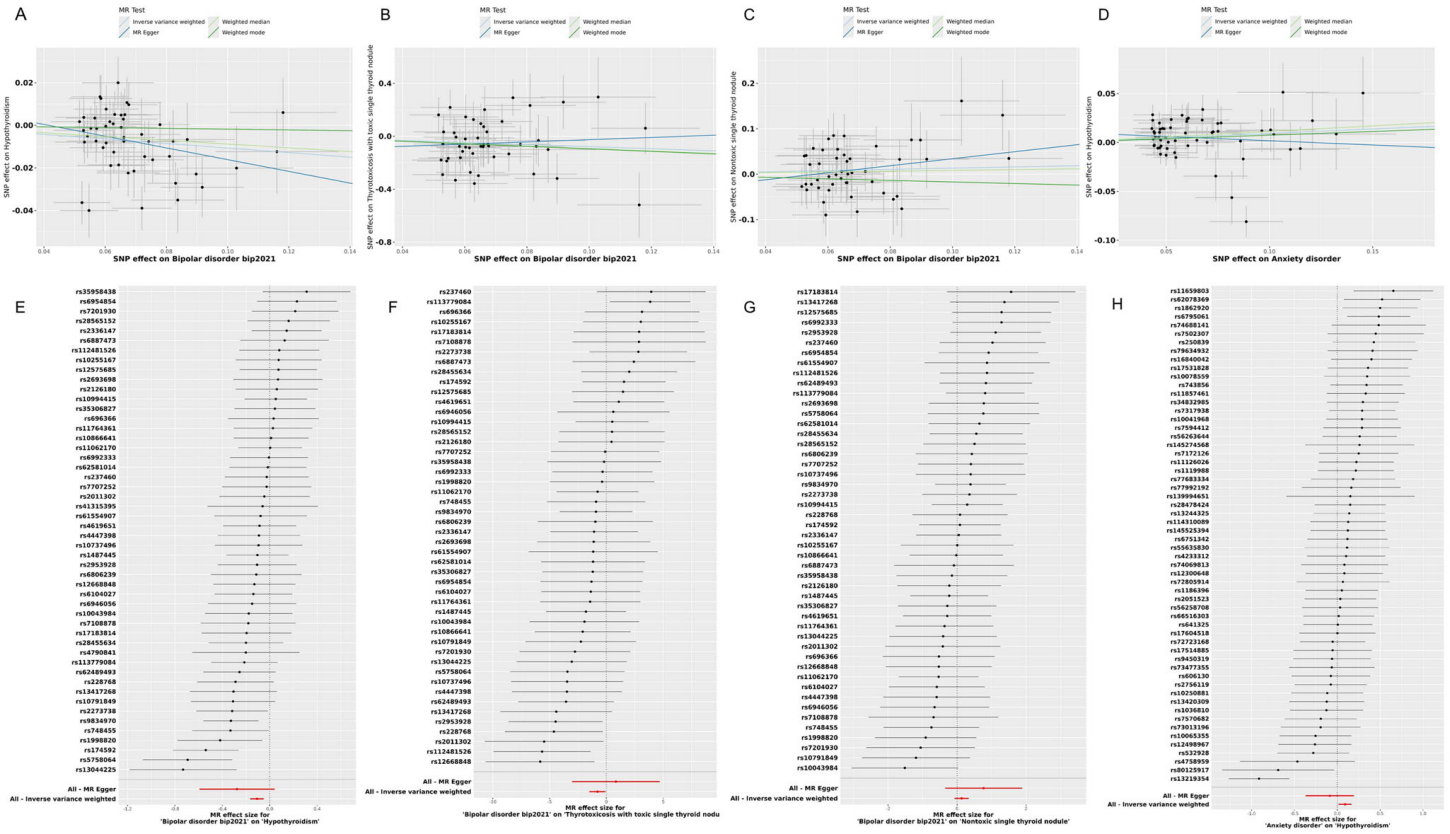
Scatter plots and forest plot for the MR analysis. (A) The scatter plot for bipolar disorder on hypothyroidism. (B) The scatter plot for bipolar disorder on thyrotoxicosis with toxic single thyroid nodule. (C) The scatter plot for bipolar disorder on non‐cancer illness code, self‐reported hyperthyroidism/thyrotoxicosis. (D) The scatter plot for anxiety disorder on hypothyroidism. (E) The forest plot for bipolar disorder on hypothyroidism. (F) The forest plot for bipolar disorder on thyrotoxicosis with toxic single thyroid nodule. (G) The forest plot for bipolar disorder on non‐cancer illness code, self‐reported hyperthyroidism/thyrotoxicosis. (H) The forest plot for anxiety disorder on hypothyroidism.

We identified potential causal evidence when studying the correlation between emotional disorders–related diseases and thyroid disorders. IVW estimates revealed negative causal relationships between bipolar disorder and hypothyroidism (OR = 0.899, 95% CI = 0.849–0.952, *p* < 0.01), toxic single thyroid nodule in thyrotoxicosis (OR = 0.455, 95% CI = 0.224–0.924, *p* = 0.029), and hyperthyroidism/toxicity (OR = 0.999, 95% CI = 0.998–1, *p* = 0.032). Additionally, there was a positive causal relationship between anxiety disorders and hypothyroidism (OR = 1.097, 95% CI = 1.016–1.183, *p* = 0.017; Table 1). Scatter plots for the relevant causal analyses are provided in Figure 2A–D, and forest plots for single SNP effect analyses are shown in Figure 2E–H. However, in the IV setting, we did not find any causal relationships between recurrent or chronic depression (*p* < 5 ×10^−5^) and severe depression (*p* < 5×10^−6^) with thyroid disorders. The results of MR analyses for all emotional disorders–related diseases are listed in Table S3.

**TABLE 1:** Mendelian randomization estimates for the relationship between emotional disorders–related diseases and thyroid disorders.

Exposure	Outcome	N.SNPs	Methods	OR (95% CI)	*p*
Bipolar disorder bip2021	Hypothyroidism	50	IVW	0.89864 (0.8486–0.95163)	<0.01
		50	MR Egger	0.75926 (0.55275–1.04292)	0.0955
		50	Weighted median	0.91566 (0.85621–0.97924)	0.0101
		50	Weighted mode	0.98202 (0.83205–1.15902)	0.83101
Bipolar disorder bip2021	Thyrotoxicosis with toxic single thyroid nodule	48	IVW	0.45547 (0.2244–0.92449)	0.02945
		48	MR Egger	2.31804 (0.04916–109.30619)	0.67092
		48	Weighted median	0.3944 (0.14447–1.07674)	0.06941
		48	Weighted mode	0.38589 (0.06116–2.43467)	0.31615
Bipolar disorder bip2021	Non‐cancer illness code, self‐reported: hyperthyroidism/thyrotoxicosis	47	IVW	0.99893 (0.99796–0.99991)	0.03176
		47	MR Egger	0.99564 (0.98897–1.00235)	0.2087
		47	Weighted median	0.99851 (0.99723–0.99979)	0.02244
		47	Weighted mode	0.99803 (0.99519–1.00088)	0.18123
Anxiety disorder	Hypothyroidism	58	IVW	1.09655 (1.01641–1.18301)	0.01729
		58	MR Egger	0.91691 (0.69161–1.2156)	0.54896
		58	Weighted median	1.11995 (1.02669–1.22168)	0.01065
		58	Weighted mode	1.07792 (0.86886–1.33727)	0.49794

We conducted pleiotropy analysis using MR‐Egger regression and quantified heterogeneity using Cochran's Q test to mitigate excessive bias.

**FIGURE 3:**
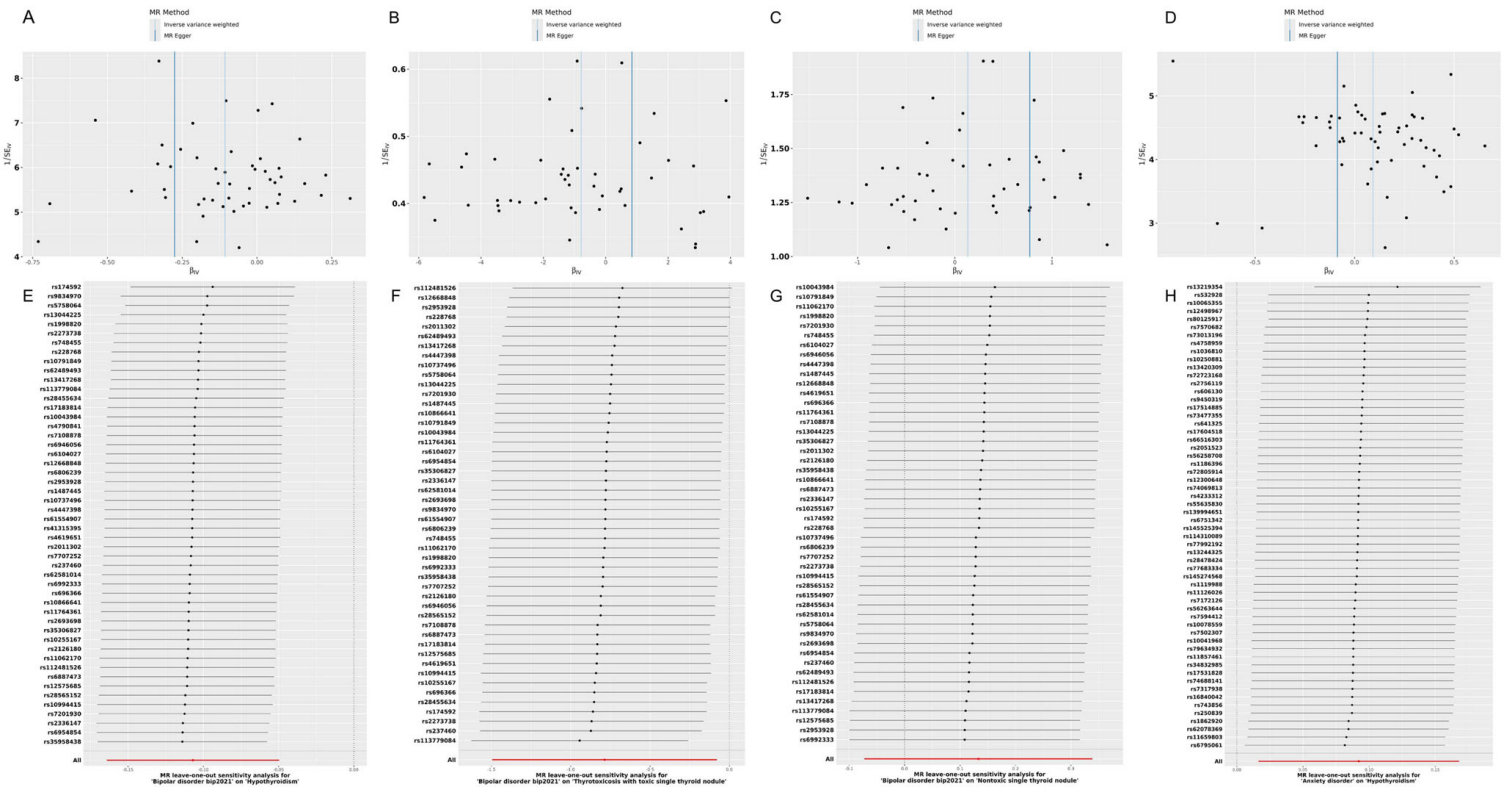
Sensitivity analysis of emotional disorders–related diseases significantly associated with thyroid disorders. (A) Funnel plots in MR analysis for bipolar disorder on hypothyroidism. (B) Funnel plots in MR analysis for bipolar disorder on thyrotoxicosis with toxic single thyroid nodule. (C) Funnel plots in MR analysis for bipolar disorder on non‐cancer illness code, self‐reported hyperthyroidism/thyrotoxicosis. (D) Funnel plots in MR analysis for anxiety disorder on hypothyroidism. (E) The “leave‐one‐out” sensitivity test for bipolar disorder on hypothyroidism. (F) The “leave‐one‐out” sensitivity test for bipolar disorder on thyrotoxicosis with toxic single thyroid nodule. (G) The “leave‐one‐out” sensitivity test for bipolar disorder on non‐cancer illness code, self‐reported hyperthyroidism/thyrotoxicosis. (H) The “leave‐one‐out” sensitivity test for anxiety disorder on hypothyroidism.

In our analysis of anxiety disorders associated with hyperthyroidism/toxicity, the results of Cochran's Q test indicate heterogeneity among some IVs for the aforementioned inflammatory factors (*p* < 0.05; Table S4). Based on the comprehensive analysis results of this study, we deemed this bias tolerable. This is because the IVW method leverages the effects of multiple genetic variants on the target variable, which can mitigate biases potentially induced by genetic variation. According to the results of the MR Egger regression and the MR‐PRESSO global test, significant horizontal pleiotropy was detected in some analyses, both showing weak positive associations influenced by one outlier (Tables S4 and S5). Leave‐one‐out analysis indicates that the causal estimates for emotional disorders–related diseases and thyroid disorders are not driven by any single SNP (Figure 3). Overall, our MR analyses are considered reliable and robust.

### 3.2 Associations of Thyroid Disorders With Emotional Disorders–Related Diseases

In this study, 67 IVs related to hypothyroidism were selected, with a mean F‐statistic of 22.428; 8 IVs related to autoimmune thyroiditis had a mean F‐statistic of 22.428; 69 IVs related to nontoxic thyroid nodules had a mean F‐statistic of 18.211; 58 IVs related to thyrotoxicosis with toxic solitary thyroid nodules had a mean F‐statistic of 18.380; 58 IVs related to hyperthyroidism/toxicity had a mean F‐statistic of 59.692; and 13 IVs related to thyroid cancer had a mean F‐statistic of 36.451. However, in the corresponding MR analysis, 61 SNPs did not match the information in the pooled data, and these unmatched SNPs could not be replaced in the results. Detailed information about the selected IVs is provided in Table S2.

**FIGURE 4:**
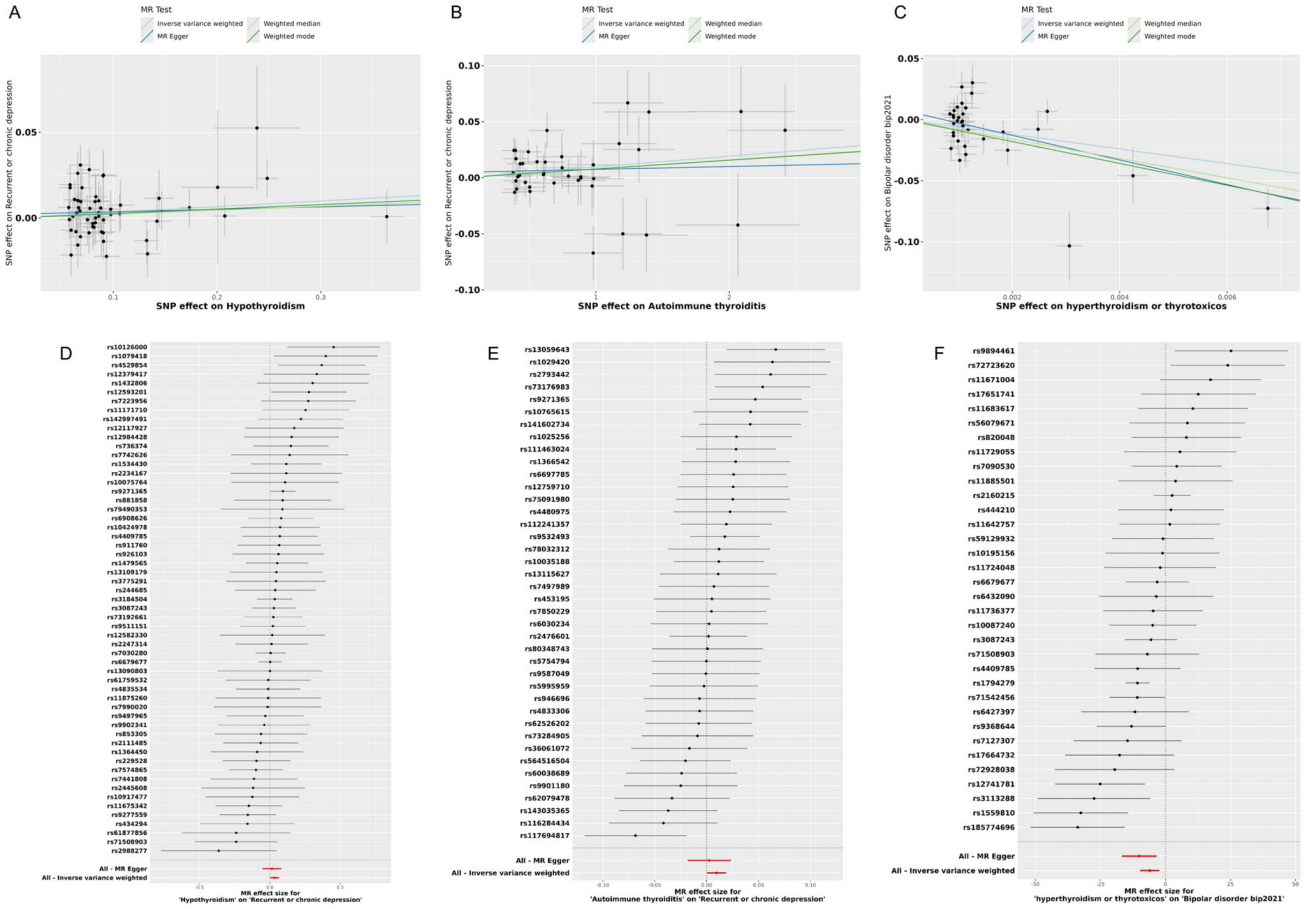
Scatter plots and forest plot for the MR analysis. (A) The scatter plot for hypothyroidism on recurrent or chronic depression. (B) The scatter plot for autoimmune thyroiditis on recurrent or chronic depression. (C) The scatter plot for hyperthyroidism/toxicity on bipolar emotional disorders. (D) The forest plot for hypothyroidism on recurrent or chronic depression. (E) The forest plot for autoimmune thyroiditis on recurrent or chronic depression. (F) The forest plot for hyperthyroidism/toxicity on bipolar emotional disorders.

We identified potential causal evidence when studying the correlation between thyroid disorders and emotional disorders–related diseases. IVW estimates revealed a positive causal relationship between hypothyroidism (OR = 1.034, 95% CI = 1.002–1.066, *p* = 0.035), autoimmune thyroiditis (OR = 1.028, 95% CI = 1.013–1.043, *p* < 0.01), and recurrent or chronic depression, and the results of other supplementary analysis methods are consistent with IVW. Additionally, there was a negative causal relationship between hyperthyroidism/toxicity and bipolar disorder (OR = 0.003, 95% CI = 0.001–0.101, *p* = 0.001) (Table 2). Scatter plots for the relevant causal analyses are provided in Figure 4A–C, and forest plots for single SNP effect analyses are shown in Figure 4D–F. The results of MR analyses for all thyroid disorders are listed in Table S3.

**TABLE 2:** Mendelian randomization estimates for the relationship between thyroid disorders and emotional disorders–related diseases.

Exposure	Outcome	N.SNPs	Methods	OR (95% CI)	*p*
Hypothyroidism	Recurrent or chronic depression	57	Inverse variance weighted	1.03367 (1.00233–1.06598)	0.03503
		57	MR Egger	1.01443 (0.9491–1.08427)	0.67475
		57	Weighted median	1.0231 (0.97683–1.07156)	0.33345
		57	Weighted mode	1.02647 (0.97316–1.0827)	0.34109
Autoimmune thyroiditis	Recurrent or chronic depression	8	Inverse variance weighted	1.02792 (1.01269–1.04338)	<0.01
		8	MR Egger	1.01317 (0.98135–1.04603)	0.45214
		8	Weighted median	1.0188 (0.99934–1.03865)	0.05842
		8	Weighted mode	1.01682 (0.99032–1.04404)	0.25566
Non‐cancer illness code, self‐reported: hyperthyroidism/thyrotoxicosis	Bipolar disorder bip2021	34	Inverse variance weighted	0.0025 (6 × 10^−5^–0.10085)	0.00149
		34	MR Egger	4 × 10^−5^ (0–0.03467)	0.00602
		34	Weighted median	0.00035 (1 × 10^−5^–0.02309)	1.9 0× 10^−4^
		34	Weighted mode	0.00013 (0–0.01209)	4.90 × 10^−4^

**FIGURE 5:**
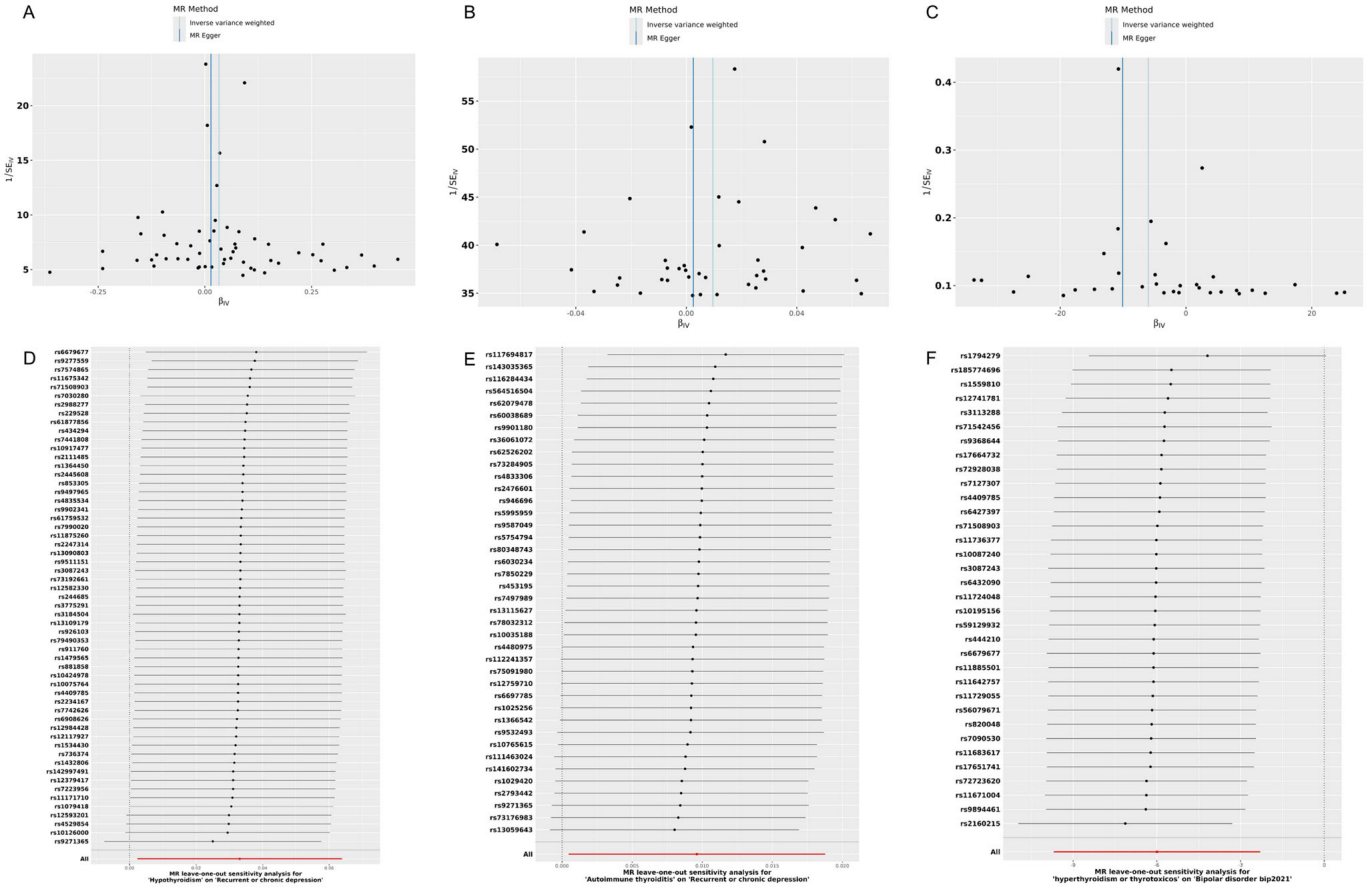
Sensitivity analysis of emotional disorders–related diseases significantly associated with thyroid disorders. (A) Funnel plots in MR analysis for hypothyroidism on recurrent or chronic depression. (B) Funnel plots in MR analysis for autoimmune thyroiditis on recurrent or chronic depression. (C) Funnel plots in MR analysis for hyperthyroidism/toxicity on bipolar emotional disorders. (D) The “leave‐one‐out” sensitivity test for hypothyroidism on recurrent or chronic depression. (E) The “leave‐one‐out” sensitivity test for autoimmune thyroiditis on recurrent or chronic depression. (F) The “leave‐one‐out” sensitivity test for hyperthyroidism/toxicity on bipolar emotional disorders.

The results of Cochran's Q test indicate heterogeneity among some IVs for the aforementioned thyroid disorders (*p* < 0.05; Table S4). Combining the results of the leave‐one‐out analysis with the comprehensive analysis, we conclude that no single SNP can influence the stability of the results. According to the results of the MR Egger regression and the MR‐PRESSO global test, significant horizontal pleiotropy was detected in some analyses (Tables S4 and S5). However, even after removing outliers, there was still no causal association between them. Leave‐one‐out analysis indicates that the causal estimates are not driven by any single SNP (Figure 5).

## 4 Discussion

This study employed a bidirectional MR approach to investigate, for the first time, the causal relationship between emotional disorders–related diseases and thyroid disorders. This innovative study design allowed for the exploration of potential associations from both directions, offering a new perspective on understanding this complex relationship. Through this method, several potential causal relationships were uncovered, providing important insights into the interplay between emotional disorders and thyroid diseases and offering implications for clinical management, thus holding substantial clinical and scientific significance.

Previous clinical research and meta‐analyses have explored the relationship between emotional disorders and thyroid dysfunction, proposing hypotheses regarding their potential mutual influence (Shen et al., 2019; Zhao et al., 2021). Some studies have reported higher prevalence rates of thyroid dysfunction among individuals with emotional disorders and vice versa, providing background and theoretical foundations for our study (Kafle, Khadka, and Tiwari 2020; Ritchie and Yeap 2015). However, certain clinical studies and meta‐analyses failed to observe significant associations between emotional disorders and thyroid diseases, suggesting the presence of some contradictions and controversies that require further investigation to clarify the relationship.

Our study revealed bidirectional causal relationships between emotional disorders and thyroid diseases. Specifically, bipolar disorder is negatively associated with hypothyroidism (OR = 0.899, 95% CI = 0.849–0.952, *p* < 0.01), toxic single thyroid nodule in thyrotoxicosis (OR = 0.455, 95% CI = 0.224–0.924, *p* = 0.029), and thyrotoxicosis/hyperthyroidism (OR = 0.999, 95% CI = 0.998–1, *p* = 0.032). Anxiety disorder is positively correlated with hypothyroidism (OR = 1.097, 95% CI = 1.016–1.183, *p* = 0.017). Additionally, hypothyroidism (OR = 1.034, 95% CI = 1.002–1.066, *p* = 0.035), autoimmune thyroiditis (OR = 1.028, 95% CI = 1.013–1.043, *p* < 0.01), and recurrent or chronic depression are positively associated. Conversely, thyrotoxicosis/hyperthyroidism is negatively associated with bipolar disorder (OR = 0.003, 95% CI = 0.001–0.101, *p* = 0.001). This indicates that the association between emotional disorders and thyroid diseases is not merely a simple correlation but rather a complex interplay between the two. Furthermore, we identified specific associations between certain types of emotional disorders and thyroid diseases. These findings suggest that different types of emotional disorders may be associated with specific types of thyroid diseases.

In exploring the underlying reasons for these findings, several perspectives can be considered. Environmental factors, such as stress, nutritional status, and lifestyle, are often associated with both emotional disorders and thyroid diseases in observational studies (Darooghegi Mofrad et al., 2019; Ruggeri et al., 2021). These factors may interact with genetic predispositions, leading to effects that are challenging to observe directly in genetic studies. Observational studies struggle to determine the direction of causality, while genetic studies, although promising in revealing causal pathways, are constrained by limitations in sample size, coverage of genetic markers, and study design. Additionally, observational studies suggest that emotional disorders and thyroid diseases may be regulated by the neuroendocrine axis (Fischer 2021; Wronski et al., 2022). Chronic anxiety or depression may affect thyroid hormone synthesis and release by influencing the hypothalamic–pituitary–thyroid axis (Dwyer et al., 2020; Mokrani et al., 2020), while abnormal thyroid hormone levels may impact neurotransmitters and neural circuits in the brain, affecting emotional regulation (Flach et al., 2021). Inflammatory responses also play a significant role in the pathogenesis of emotional disorders and thyroid diseases (Cai et al., 2018; Yao et al., 2023). Immune‐mediated thyroid diseases, such as autoimmune thyroiditis, may induce anxiety or depression symptoms through inflammatory pathways, while emotional disorders may lead to chronic inflammation, affecting thyroid function (Jurado‐Flores, Warda, and Mooradian 2022; Valencia‐Sanchez et al., 2021). However, given the numerous subtypes of emotional disorders and thyroid diseases, each with potentially distinct pathophysiological mechanisms, future research needs to carefully differentiate disease subtypes to accurately elucidate their relationships.

Our study has several strengths, including the use of MR methodology, leveraging genetic variants as IVs, and conducting sensitivity analyses to minimize confounding and reverse causation bias. The bidirectional design of our study allowed for a comprehensive exploration of the causal relationships between emotional disorders–related diseases and thyroid diseases, enhancing the reliability of our findings. This study met all three MR assumptions. In the present study, genetic IVs were selected based on their significant association with the exposures (emotional disorders or thyroid disorders), using rigorous genome‐wide significance thresholds (e.g., *p* < 5 × 10^−8^), but when too few SNPs were available, less stringent thresholds were applied (*p* < 5 × 10^−6^) (Xu et al., 2023). The relevance of these IVs was further validated by calculating the F‐statistics, which were consistently above 10, ensuring that the weak instrumental bias was minimized. LD pruning was performed to ensure independence between selected SNPs. Furthermore, potential horizontal pleiotropy was examined using the MR‐Egger intercept tests and MR‐PRESSO. The results demonstrated no significant horizontal pleiotropy in most analyses, indicating that the IVs are unlikely to be associated with confounders. Finally, by design, MR ensures that the exclusivity assumption is inherently supported when the IVs are selected from GWAS with no direct association with the outcome other than through exposure. Sensitivity analyses, such as Cochran's Q test and leave‐one‐out analyses, confirmed that no single SNP disproportionately influenced the outcomes, supporting the exclusivity assumption.

However, our study also has limitations that need to be considered. Considering the quality of genetic data, sample size, and comprehensiveness of research resources, we selected some exposure and outcome data from the same database to facilitate the discovery of stronger genetic association signals. However, this approach may lead to collinearity issues, sample homogeneity limitations, and potential bias risks. To minimize these effects and ensure the robustness of our findings, we implemented various sensitivity analyses, such as the MR‐Egger and MR‐PRESSO methods. Our results are also consistent with previous clinical and mechanistic studies. Future research can use different databases or independent cohorts to replicate the analysis and further validate our current findings.

In summary, our bidirectional MR study provides evidence of potential causal relationships between emotional disorders–related diseases and thyroid diseases. These findings preliminarily explore the relationship between emotional disorder–related diseases and thyroid diseases, laying the groundwork for future research to further investigate the biological mechanisms underlying this correlation. Exploring the roles of specific hormone pathways, immune responses, and gut microbiota in emotional regulation and thyroid function may elucidate common pathophysiological mechanisms.

## 5 Conclusion

This study reveals bidirectional causal associations between genetically predicted emotional disorders and genetically predicted thyroid diseases, alongside specific associations between certain types of emotional disorders and distinct thyroid conditions. Nevertheless, such findings need further validation through additional research. In addition, clinical studies are required to explore the potential biological mechanisms underlying and linking these diseases.

## Author Contributions

**Jiaying Fan**: Conceptualization, project administration, data curation, formal analysis, investigation, methodology, writing–original draft, writing–review and editing, resources. **Kai Zhou**: Project administration, writing–original draft, writing–review and editing, resources. **Cuiwen Yu**: Investigation, methodology, formal analysis, data curation, writing–review and editing, writing–original draft.

## Ethics Statement

This article is a Mendelian randomization study. The data come from an online database and do not require ethical approval.

## Consent

This article is a Mendelian randomization study. The data come from an online database and do not require written informed consent.

## Conflicts of Interest

The authors declare no conflicts of interest.

### Peer Review

The peer review history for this article is available at https://publons.com/publon/10.1002/brb3.70252.

## Supporting information



Table S1 GWAS information for all outcomes and exposures.

Table S2 Detailed information of instrumental variables used in MR analyses

Table S3 MR analysis of all exposure on the outcome

Table S4 The heterogeneity and pleiotropy results

Table S5 The MR pleiotropy residual sum and outlier results

## Data Availability

All data generated or analyzed during this study are included in this article and supplementary information files.

## References

[brb370252-bib-0001] Bajaj, J. K. , P. Salwan , and S. Salwan . 2016. “Various Possible Toxicants Involved in Thyroid Dysfunction: A Review.” J Clin Diagn Res 10, no. 1: Fe01–03. 10.7860/jcdr/2016/15195.7092. PMC4740614 26894086

[brb370252-bib-0002] Bao, L. , Z. Wang , L. Wu , Z. Luo , and Y. Wang . 2024. “Gut Microbiota's Influence on Erysipelas: Evidence From a Two‐Sample Mendelian Randomization Analysis.” Frontiers in Cellular and Infection Microbiology 14: 1371591. 10.3389/fcimb.2024.1371591. 38638831 PMC11024262

[brb370252-bib-0003] Bowden, J. , G. Davey Smith , P. C. Haycock , and S. Burgess . 2016. “Consistent Estimation in Mendelian Randomization With Some Invalid Instruments Using a Weighted Median Estimator.” Genetic Epidemiology 40, no. 4: 304–314. 10.1002/gepi.21965. 27061298 PMC4849733

[brb370252-bib-0004] Bowden, J. , and M. V. Holmes . 2019. “Meta‐Analysis and Mendelian Randomization: A Review.” Research Synthesis Methods 10, no. 4: 486–496. 10.1002/jrsm.1346. 30861319 PMC6973275

[brb370252-bib-0005] Burgess, S. , and S. G. Thompson . 2017. “Interpreting Findings From Mendelian Randomization Using the MR‐Egger Method.” European Journal of Epidemiology 32, no. 5: 377–389. 10.1007/s10654-017-0255-x. 28527048 PMC5506233

[brb370252-bib-0006] Cai, Y. J. , F. Wang , Z. X. Chen , et al. 2018. “Hashimoto's Thyroiditis Induces Neuroinflammation and Emotional Alterations in Euthyroid Mice.” J Neuroinflammation 15, no. 1: 299. 10.1186/s12974-018-1341-z. 30373627 PMC6206655

[brb370252-bib-0007] Chen, L. G. , J. D. Tubbs , Z. Liu , T. Q. Thach , and P. C. Sham . 2024. “Mendelian Randomization: Causal Inference Leveraging Genetic Data.” Psychological Medicine 54, no. 8: 1461–1474. 10.1017/s0033291724000321. 38639006

[brb370252-bib-0008] Connelly, K. J. , J. J. Park , and S. H. LaFranchi . 2022. “History of the Thyroid.” Horm Res Paediatr 95, no. 6: 546–556. 10.1159/000526621. 36446327

[brb370252-bib-0009] Darooghegi Mofrad, M. , F. Siassi , B. Guilani , N. Bellissimo , and L. Azadbakht . 2019. “Association of Dietary Phytochemical Index and Mental Health in Women: A Cross‐Sectional Study.” British Journal of Nutrition 121, no. 9: 1049–1056. 10.1017/s0007114519000229. 30714542

[brb370252-bib-0010] Davies, N. M. , M. V. Holmes , and G. Davey Smith . 2018. “Reading Mendelian Randomisation Studies: A Guide, Glossary, and Checklist for Clinicians.” Bmj 362: k601. 10.1136/bmj.k601. 30002074 PMC6041728

[brb370252-bib-0011] Dehn, L. B. , and T. Beblo . 2019. “Depressed, Biased, Forgetful: The Interaction of Emotional and Cognitive Dysfunctions in Depression.” Neuropsychiatr 33, no. 3: 123–130. 10.1007/s40211-019-0307-4 (Verstimmt, verzerrt, vergesslich: Das Zusammenwirken emotionaler und kognitiver Dysfunktionen bei Depression.). 30875025

[brb370252-bib-0012] Dwyer, J. B. , A. Aftab , R. Radhakrishnan , et al. 2020. “Hormonal Treatments for Major Depressive Disorder: State of the Art.” American Journal of Psychiatry 177, no. 8: 686–705. 10.1176/appi.ajp.2020.19080848. 32456504 PMC7841732

[brb370252-bib-0013] Fischer, S. 2021. “The Hypothalamus in Anxiety Disorders.” Handb Clin Neurol 180: 149–160. 10.1016/b978-0-12-820107-7.00009-4. 34225926

[brb370252-bib-0014] Flach, E. , J. Koenig , P. van der Venne , P. Parzer , F. Resch , and M. Kaess . 2021. “Hypothalamic‐Pituitary‐Thyroid Axis Function in Female Adolescent Nonsuicidal Self‐Injury and Its Association With Comorbid Borderline Personality Disorder and Depression.” Progress in Neuro‐Psychopharmacology & Biological Psychiatry 111: 110345. 10.1016/j.pnpbp.2021.110345. 33964324

[brb370252-bib-0015] Gorkhali, B. , S. Sharma , M. Amatya , D. Acharya , and M. Sharma . 2020. “Anxiety and Depression Among Patients With Thyroid Function Disorders.” Journal of Nepal Health Research Council 18, no. 3: 373–378. 10.33314/jnhrc.v18i3.2499. 33210626

[brb370252-bib-0016] Hartwig, F. P. , G. Davey Smith , and J. Bowden . 2017. “Robust Inference in Summary Data Mendelian Randomization via the Zero Modal Pleiotropy Assumption.” International Journal of Epidemiology 46, no. 6: 1985–1998. 10.1093/ije/dyx102. 29040600 PMC5837715

[brb370252-bib-0017] Haycock, P. C. , S. Burgess , K. H. Wade , J. Bowden , C. Relton , and G. Davey Smith . 2016. “Best (but oft‐forgotten) Practices: The Design, Analysis, and Interpretation of Mendelian Randomization Studies.” American Journal of Clinical Nutrition 103, no. 4: 965–978. 10.3945/ajcn.115.118216. 26961927 PMC4807699

[brb370252-bib-0018] He, B. , Q. Lyu , L. Yin , M. Zhang , Z. Quan , and Y. Ou . 2021. “Depression and Osteoporosis: A Mendelian Randomization Study.” Calcified Tissue International 109, no. 6: 675–684. 10.1007/s00223-021-00886-5. 34259888 PMC8531056

[brb370252-bib-0019] Ji, D. , W. Z. Chen , L. Zhang , Z. H. Zhang , and L. J. Chen . 2024. “Gut Microbiota, Circulating Cytokines and Dementia: A Mendelian Randomization Study.” J Neuroinflammation 21, no. 1: 2. 10.1186/s12974-023-02999-0. 38178103 PMC10765696

[brb370252-bib-0020] Jurado‐Flores, M. , F. Warda , and A. Mooradian . 2022. “Pathophysiology and Clinical Features of Neuropsychiatric Manifestations of Thyroid Disease.” Journal of the Endocrine Society 6, no. 2: bvab194. 10.1210/jendso/bvab194. 35059548 PMC8765786

[brb370252-bib-0021] Kafle, B. , B. Khadka , and M. L. Tiwari . 2020. “Prevalence of Thyroid Dysfunction Among Depression Patients in a Tertiary Care Centre.” JNMA Journal of Nepal Medical Association 58, no. 229: 654–658. 10.31729/jnma.5296. PMC7580338 33068085

[brb370252-bib-0022] Li, H. , M. Li , S. Dong , S. Zhang , A. Dong , and M. Zhang . 2023. “Assessment of the Association Between Genetic Factors Regulating Thyroid Function and Microvascular Complications in Diabetes: A Two‐Sample Mendelian Randomization Study in the European Population.” Front Endocrinol (Lausanne) 14: 1126339. 10.3389/fendo.2023.1126339. 36926020 PMC10011638

[brb370252-bib-0023] Mariani, G. , M. Tonacchera , M. Grosso , et al. 2021. “The Role of Nuclear Medicine in the Clinical Management of Benign Thyroid Disorders, Part 2: Nodular Goiter, Hypothyroidism, and Subacute Thyroiditis.” Journal of Nuclear Medicine 62, no. 7: 886–895. 10.2967/jnumed.120.251504. 33579801

[brb370252-bib-0024] Mokrani, M. C. , F. Duval , A. Erb , F. Gonzalez Lopera , and V. Danila . 2020. “Are the Thyroid and Adrenal System Alterations Linked in Depression?” Psychoneuroendocrinology 122: 104831. 10.1016/j.psyneuen.2020.104831. 33068950

[brb370252-bib-0025] Nuñez, N. A. , B. Joseph , M. Pahwa , et al. 2022. “Augmentation Strategies for Treatment Resistant Major Depression: A Systematic Review and Network Meta‐Analysis.” Journal of Affective Disorders 302: 385–400. 10.1016/j.jad.2021.12.134. 34986373 PMC9328668

[brb370252-bib-0026] Ritchie, M. , and B. B. Yeap . 2015. “Thyroid Hormone: Influences on Mood and Cognition in Adults.” Maturitas 81, no. 2: 266–275. 10.1016/j.maturitas.2015.03.016. 25896972

[brb370252-bib-0027] Ruggeri, R. M. , S. Giovinazzo , M. C. Barbalace , et al. 2021. “Influence of Dietary Habits on Oxidative Stress Markers in Hashimoto's Thyroiditis.” Thyroid: Official Journal of the American Thyroid Association 31, no. 1: 96–105. 10.1089/thy.2020.0299. 32729374

[brb370252-bib-0028] Sanderson, E. , W. Spiller , and J. Bowden . 2021. “Testing and Correcting for Weak and Pleiotropic Instruments in Two‐Sample Multivariable Mendelian Randomization.” Statistics in Medicine 40, no. 25: 5434–5452. 10.1002/sim.9133. 34338327 PMC9479726

[brb370252-bib-0029] Shen, Y. , F. Wu , Y. Zhou , et al. 2019. “Association of Thyroid Dysfunction With Suicide Attempts in First‐Episode and Drug Naïve Patients With Major Depressive Disorder.” Journal of Affective Disorders 259: 180–185. 10.1016/j.jad.2019.08.067. 31446378

[brb370252-bib-0030] Valencia‐Sanchez, C. , S. J. Pittock , C. Mead‐Harvey , et al. 2021. “Brain Dysfunction and Thyroid Antibodies: Autoimmune Diagnosis and Misdiagnosis.” Brain Communications 3, no. 2: fcaa233. 10.1093/braincomms/fcaa233. 34061124 PMC8152924

[brb370252-bib-0031] Verbanck, M. , C. Y. Chen , B. Neale , and R. Do . 2018. “Detection of Widespread Horizontal Pleiotropy in Causal Relationships Inferred From Mendelian Randomization Between Complex Traits and Diseases.” Nature Genetics 50, no. 5: 693–698. 10.1038/s41588-018-0099-7. 29686387 PMC6083837

[brb370252-bib-0032] Wang, X. , X. Wang , H. Wang , M. Yang , W. Dong , and D. Shao . 2023. “Association Between Psoriasis and Lung Cancer: Two‐Sample Mendelian Randomization Analyses.” BMC Pulmonary Medicine 23, no. 1: 4. 10.1186/s12890-022-02297-0. 36604675 PMC9814449

[brb370252-bib-0033] Wei, T. , Z. Zhu , L. Liu , et al. 2023. “Circulating Levels of Cytokines and Risk of Cardiovascular Disease: A Mendelian Randomization Study.” Frontiers in immunology 14: 1175421. 10.3389/fimmu.2023.1175421. 37304261 PMC10247976

[brb370252-bib-0034] Wronski, M. L. , F. I. Tam , M. Seidel , et al. 2022. “Associations Between Pituitary‐Thyroid Hormones and Depressive Symptoms in Individuals With Anorexia Nervosa Before and After Weight‐Recovery.” Psychoneuroendocrinology 137: 105630. 10.1016/j.psyneuen.2021.105630. 34959165

[brb370252-bib-0035] Xu, F. , X. Gan , Y. Tao , et al. 2023. “Association Between Gut Microbiota and Influenza: A Bidirectional Two‐Sample Mendelian Randomization Study.” BMC Infectious Diseases [Electronic Resource] 23, no. 1: 692. 10.1186/s12879-023-08706-x. 37848822 PMC10580584

[brb370252-bib-0036] Yao, H. , D. Zhang , H. Yu , et al. 2023. “Gut Microbiota Regulates Chronic Ethanol Exposure‐Induced Depressive‐Like Behavior Through Hippocampal NLRP3‐Mediated Neuroinflammation.” Molecular Psychiatry 28, no. 2: 919–930. 10.1038/s41380-022-01841-y. 36280756 PMC9908543

[brb370252-bib-0037] Zhang, X. , X. Wang , H. Hu , et al. 2024. “Prevalence of Self‐Reported Thyroid Disease Among Adults With Depression.” Journal of Psychosomatic Research 176: 111557. 10.1016/j.jpsychores.2023.111557. 38056108

[brb370252-bib-0038] Zhao, S. , X. Zhang , Y. Zhou , et al. 2021. “Comparison of Thyroid Function in Different Emotional States of Drug‐Naïve Patients With Bipolar Disorder.” BMC Endocrine Disorders 21, no. 1: 210. 10.1186/s12902-021-00869-5. 34674686 PMC8532266

