# Supplementary material for: Causal Relationship Between Emotional Disorders and Thyroid Disorders: A Bidirectional Two‐Sample Mendelian Randomization Study
Source: Brain Behav. 2025 Jan 19;15(1):e70252. doi: 10.1002/brb3.70252 (PMC11743992; doi:10.1002/brb3.70252)
Supplement: Supplementary file 1 — Table S1 GWAS information for all outcomes and exposures. [file BRB3-15-e70252-s004.docx]

**Table S1. GWAS information for all outcomes and exposures**

| **Trait** | **GWAS ID** | **sample (case/control)** | **SNPs number** |
| --- | --- | --- | --- |
| Hypothyroidism | ebi-a-GCST90018862 | 379,986/30,155 | 24138872 |
| Autoimmune thyroiditis | finn-b-E4_THYROIDITAUTOIM | 187,684/244 | 16380358 |
| Nontoxic single thyroid nodule | finn-b-E4_GOITRENOD | 187,684/1,121 | 16380359 |
| Thyrotoxicosis with toxic single thyroid nodule | finn-b-E4_THYTOXNOD | 214,650/110 | 16380459 |
| Non-cancer illness code, self-reported: hyperthyroidism/thyrotoxicosis | ukb-b-20289 | 459,388/3,545 | 9851867 |
| Thyroid cancer | ebi-a-GCST90018929 | 490,920/1,054 | 24198226 |
| Major depressive disorder | ebi-a-GCST90086058 | 49,373/7,264 | 14426915 |
| Bipolar disorder bip2021 | ieu-b-5110 | 371,549/41,917 | / |
| Recurrent or chronic depression | F5_DEPRESSION_RECURRENT | 19,388/246,043 | / |
| Anxiety disorder | F5_ALLANXIOUS | 27,664/368,054 | / |
