# Supplementary material for: Causal Relationship Between Emotional Disorders and Thyroid Disorders: A Bidirectional Two‐Sample Mendelian Randomization Study
Source: Brain Behav. 2025 Jan 19;15(1):e70252. doi: 10.1002/brb3.70252 (PMC11743992; doi:10.1002/brb3.70252)
Supplement: Supplementary file 4 — Table S4 The heterogeneity and pleiotropy results [file BRB3-15-e70252-s001.docx]

**Table S4. The heterogeneity and pleiotropy results**

| **Exposure** | **Outcome** | **Heterogeneity** | | **Pleiotropy** | |
| --- | --- | --- | --- | --- | --- |
|  |  | **Q statistic (IVW)** | ***P* value** | **MR-Egger Intercept** | ***P* value** |
| Major depressive disorder | Hypothyroidism | 21.98800567 | 0.055547899 | -0.012744185 | 0.45264724 |
|  | Autoimmune thyroiditis | 16.17424854 | 0.094751801 | -0.198772426 | 0.21845844 |
|  | Nontoxic single thyroid nodule | 13.94727045 | 0.175410383 | -0.135754185 | 0.042268916 |
|  | Thyrotoxicosis with toxic single thyroid nodule | 5.678924123 | 0.841478844 | 0.015750807 | 0.931783971 |
|  | Non-cancer illness code, self-reported: hyperthyroidism/thyrotoxicosis | 1.893565311 | 0.755326958 | -2.22E-04 | 0.879110918 |
|  | Autoimmune thyroiditis | 16.17424854 | 0.094751801 | -0.198772426 | 0.21845844 |
|  | Thyroid cancer | 8.149572254 | 0.833721141 | 0.009435043 | 0.856430583 |
| Bipolar disorder | Hypothyroidism | 61.79219 | 0.08717868 | 0.0176914 | 0.08848434 |
|  | Autoimmune thyroiditis | 47.07427 | 0.469519505 | -0.040567047 | 0.62083229 |
|  | Nontoxic single thyroid nodule | 43.46553433 | 0.619757554 | -0.04315215 | 0.258888426 |
|  | Thyrotoxicosis with toxic single thyroid nodule | 57.32016772 | 0.143922223 | -0.10995845 | 0.4041156 |
|  | Non-cancer illness code, self-reported: hyperthyroidism/thyrotoxicosis | 55.51234524 | 0.158886483 | 2.17E-04 | 0.335269635 |
|  | Autoimmune thyroiditis | 47.07427 | 0.469519505 | -0.040567047 | 0.62083229 |
|  | Thyroid cancer | 38.34788111 | 0.863672152 | 0.013772531 | 0.687026432 |
| Recurrent or chronic depression | Hypothyroidism | 13.17362654 | 0.282128955 | -0.004709541 | 0.850166718 |
|  | Autoimmune thyroiditis | 11.1593297 | 0.430013356 | 0.059626725 | 0.714984521 |
|  | Nontoxic single thyroid nodule | 11.88910491 | 0.372036367 | 0.099599973 | 0.188855989 |
|  | Thyrotoxicosis with toxic single thyroid nodule | 15.75491649 | 0.150476155 | 0.221668289 | 0.428733166 |
|  | Non-cancer illness code, self-reported: hyperthyroidism/thyrotoxicosis | 5.578684104 | 0.781231692 | 4.73E-04 | 0.396082265 |
|  | Autoimmune thyroiditis | 11.1593297 | 0.430013356 | 0.059626725 | 0.714984521 |
|  | Thyroid cancer | 7.436029305 | 0.762754185 | 0.047376963 | 0.55451747 |
| Anxiety disorder | Hypothyroidism | 60.95273 | 0.3024174 | 0.003006409 | 0.6627599 |
|  | Autoimmune thyroiditis | 65.0432509 | 0.217009269 | -0.015359038 | 0.80058975 |
|  | Nontoxic single thyroid nodule | 46.01397529 | 0.850849994 | -0.005146348 | 0.845731418 |
|  | Thyrotoxicosis with toxic single thyroid nodule | 72.58556361 | 0.079901717 | -0.039643976 | 0.673116552 |
|  | Non-cancer illness code, self-reported: hyperthyroidism/thyrotoxicosis | 154.0654577 | 5.33E-1 5 | 0.001077111 | 7.14E-04 |
|  | Autoimmune thyroiditis | 65.0432509 | 0.217009269 | -0.015359038 | 0.80058975 |
|  | Thyroid cancer | 53.75095073 | 0.560473089 | -0.00252878 | 0.920435351 |
| Hypothyroidism | Major depressive disorder | 89.32632632 | 0.003076973 | 0.001357436 | 0.900394076 |
|  | Bipolar disorder | 86.71620962 | 0.005291538 | 0.011688396 | 0.002470759 |
|  | Recurrent or chronic depression | 56.55197288 | 0.454229659 | 0.002226681 | 0.534862602 |
|  | Anxiety disorder | 60.83100844 | 0.306220606 | 0.003484701 | 0.265175031 |
| Autoimmune thyroiditis | Major depressive disorder | 6.015271721 | 0.537967284 | -0.012418126 | 0.69550504 |
|  | Bipolar disorder | 11.88926341 | 0.104259816 | -0.023512421 | 0.224116979 |
|  | Recurrent or chronic depression | 7.379316855 | 0.390481603 | 0.014827141 | 0.35413072 |
|  | Anxiety disorder | 11.41301555 | 0.121590619 | -0.006784141 | 0.695913295 |
| Nontoxic single thyroid nodule | Major depressive disorder | 37.30268589 | 0.979803156 | -0.002036985 | 0.872103572 |
|  | Bipolar disorder | 59.16236648 | 0.326173835 | -7.35E-04 | 0.895998918 |
|  | Recurrent or chronic depression | 53.44541089 | 0.712316119 | -0.003351731 | 0.560888411 |
|  | Anxiety disorder | 70.68686526 | 0.162787755 | 0.002421045 | 0.648427199 |
| Thyrotoxicosis with toxic single thyroid nodule | Major depressive disorder | 71.33516398 | 0.00566002 | -0.003962047 | 0.800396343 |
|  | Bipolar disorder | 63.70095197 | 0.021727142 | -0.00761096 | 0.216962941 |
|  | Recurrent or chronic depression | 57.07623721 | 0.173416302 | 0.00503732 | 0.39211702 |
|  | Anxiety disorder | 56.88715 | 0.1530022 | 0.003300889 | 0.5114918 |
| Non-cancer illness code, self-reported: hyperthyroidism/thyrotoxicosis | Major depressive disorder | 32.94930084 | 0.420442589 | 0.004088481 | 0.679045984 |
|  | Bipolar disorder | 75.05755251 | 4.06E-05 | 0.007323712 | 0.165604881 |
|  | Recurrent or chronic depression | 41.06940888 | 0.067911342 | -0.00741733 | 0.355985632 |
|  | Anxiety disorder | 27.85141044 | 0.525868118 | 4.48E-04 | 0.936785524 |
| Thyroid cancer | Major depressive disorder | 9.465745937 | 0.395437999 | -5.28E-04 | 0.987580441 |
|  | Bipolar disorder | 13.38505742 | 0.145942732 | -0.021711201 | 0.140131046 |
|  | Recurrent or chronic depression | 16.85436745 | 0.051046013 | 0.009425129 | 0.594854277 |
|  | Anxiety disorder | 8.834476088 | 0.452691834 | 0.008090855 | 0.450979937 |
